# The Role of Swine Gut Microbiota and Its Metabolites in Maintaining Intestinal Barrier Integrity and Mitigating Stress via the Gut–Brain Axis

**DOI:** 10.3390/ani15243653

**Published:** 2025-12-18

**Authors:** Katarzyna Woś, Karol Pachciński, Marianna Wacko, Oliwia Koszła, Przemysław Sołek, Anna Czech

**Affiliations:** 1 Department of Biochemistry and Toxicology, University of Life Sciences in Lublin, Akademicka 13, 20-950 Lublin, Poland; kpachcinski@gmail.com (K.P.); wacko.marianna@gmail.com (M.W.); pp.solek@gmail.com (P.S.); 2 Department of Biopharmacy, Medical University of Lublin, Chodźki 4a, 20-093 Lublin, Poland; koszlaoliwia@gmail.com

**Keywords:** animal welfare, gut–brain axis, microbiota, pigs, microbial metabolites, stress

## Abstract

The gut-brain axis is a complex communication network linking the gastrointestinal tract and the central nervous system, in which the gut microbiota plays a pivotal role in regulating intestinal homeostasis, immune responses and neuroendocrine functions. This review summarizes current knowledge on the role of the porcine gut microbiota in the functioning of the gut-brain axis and examines nutritional strategies aimed at its modulation. Key production-related stressors, such as weaning, transport and handling, disrupt microbiota composition, increase intestinal permeability and activate the hypothalamic-pituitary-adrenal (HPA) axis, leading to heightened stress responses, impaired immunity and behavioral disturbances. Evidence indicates that supplementation with probiotics, prebiotics, or postbiotics stabilizes the gut microbiota, enhances the production of bioactive metabolites, supports intestinal barrier integrity and alleviates oxidative stress. Such interventions improve adaptation to environmental stress, animal welfare and performance, while potentially reducing the need for antibiotics. Maintaining a balanced gut microbiota is therefore essential for the proper functioning of the neuroendocrine and immune systems in pigs. An integrated approach utilizing omics technologies (metagenomics, metabolomics, proteomics) may further elucidate microbiota-brain interactions and support the development of sustainable and ethical swine production strategies.

## Simple Summary

The gut-brain axis is a key communication system between the intestines and the brain, regulating stress responses, immunity and animal behavior. In pigs, its functioning is particularly important due to the relatively permeable intestinal barrier and the role of the gut microbiota in maintaining homeostasis. This review discusses the physiological and molecular mechanisms of the gut-brain axis, including neural, hormonal and immune signaling, as well as the involvement of microbiota-derived metabolites, such as neurotransmitters, short-chain fatty acids, bile acids and tryptophan derivatives. Nutritional interventions are also described, which, by modulating the microbiota, can help reduce stress, improve animal welfare and support sustainable pig production.

## 1. Introduction

The assessment of pig welfare encompasses both physiological and behavioral indicators, reflecting the level of stress and the animals’ ability to adapt to environmental conditions. In pigs, production-related stressors, such as weaning, farrowing, transport or slaughter, elicit physiological and behavioral responses, including increased activity of the hypothalamic-pituitary-adrenal (HPA) axis and alterations in social and exploratory behaviors [1]. Both acute high-intensity stress and chronic stress can lead to impaired immune function, reduced intestinal barrier integrity, decreased weight gain and increased susceptibility to disease, directly affecting pig welfare and production performance [2].

In recent years, the gut-brain axis (GBA), a complex network of neuroendocrine, immune and metabolic communication between the gastrointestinal tract and the central nervous system, has been increasingly recognized as a key regulator of stress responses [3,4]. It is now understood that the effects of gut microbiota depend not only on its taxonomic composition but also on its metabolic activity [5]. Microbial metabolites play a central role in gut-brain communication by influencing the host’s immune, metabolic and neuronal processes [6].

Intestinal barrier integrity is critical for maintaining homeostasis and health in pigs. Microbial metabolites, such as short-chain fatty acids (SCFAs), tryptophan metabolites and secondary bile acids, support barrier function by regulating tight junction protein expression and modulating inflammatory responses. Disruption of barrier integrity increases intestinal permeability, promoting inflammation, behavioral disturbances and reduced immunity, which in turn can affect GBA function as well as pig welfare and production performance [7].

SCFAs, produced through the fermentation of structural carbohydrates, are particularly important, as they enhance barrier integrity, modulate inflammatory responses and regulate neuronal plasticity by influencing the expression of neurotrophins such as brain-derived neurotrophic factor (BDNF). They also activate GPR41 and GPR43 receptors and affect pathways controlling serotonin secretion and enteric nervous system (ENS) function, representing a crucial link in gut-brain signaling [6,8]. Similarly, tryptophan metabolites, including indoles, serotonin and kynurenine pathway derivatives, affect immune function, epithelial integrity and neurotransmission. During inflammation, tryptophan metabolism shifts toward the kynurenine pathway, leading to the formation of neuroactive compounds that may disrupt stress responses [8,9,10]. Microbiota-derived neurotransmitters, such asγ-aminobutyricacid (GABA), dopamine (DA), serotonin (5-HT) and noradrenaline (NA), also play key signaling roles in the ENS and, through activation of vagal afferent fibers, contribute to modulation of the gut-brain axis, influencing gut motility, behavior and stress responses [11]. Secondary bile acids, generated by bacterial conversion of primary bile acids, complement these mechanisms by activating FXR and TGR5 receptors, thereby regulating metabolism, inflammation and barrier function [12].

Microbiota-derived neurotransmitters such as GABA, DA, 5-HT and NA also represent an important component of gut signaling and their synthesis and availability depend on the composition of the gut microbiota [11]. In pigs, these compounds act as signaling molecules within the ENS and, through activation of vagal afferent fibers, contribute to modulation of the GBA, influencing gut motility, behavioral responses and stress regulation. Complementary mechanisms involve secondary bile acids, produced through bacterial transformation of primary bile acids, which activate FXR and TGR5 receptors and regulate metabolism, inflammation and intestinal barrier function [12].

Disruptions in gut microbiota composition caused by environmental stress, infections, or dietary changes can alter the profile of metabolites, including SCFAs, neurotransmitters and tryptophan derivatives. These changes may impair gut-brain axis function, affecting behavior, immune responses and stress regulation in pigs and consequently contributing to behavioral disorders and reduced immunity [7]. Therefore, understanding the mechanisms by which microbial metabolites influence neuroendocrine, immune and behavioral responses in pigs is essential for developing targeted strategies to improve welfare. Identification of key metabolites and biological pathways involved in stress regulation can inform the design of nutritional interventions, including prebiotics, probiotics and postbiotics.

Although the role of the gut microbiota in modulating emotions, anxiety-like behaviors and cognitive functions is well documented in humans and rodents, knowledge of these mechanisms in pigs remains limited. To date, research has primarily focused on the influence of gut microbiota on growth, immunity and gastrointestinal health, while its potential involvement in gut-brain communication has been explored far less extensively [1,13]. Nevertheless, due to their substantial physiological, neuroanatomical and metabolic similarities to humans and rodents, pigs represent a valuable translational model for studying interactions between the gut microbiota, the nervous system and behavior [11,14].

The aim of this review is to critically summarize the current state of knowledge on the pig gut microbiota and its metabolites in the context of intestinal barrier integrity and gut-brain axis functioning, drawing on mechanisms described in humans and rodents that may provide valuable insights for formulating hypotheses in pigs and for developing targeted microbiota-modulating nutritional strategies.

## 2. Materials and Methods

A literature search was conducted in PubMed, Scopus and Web of Science for English-language, peer-reviewed articles published between January 2015 and 9 December 2025. The search combined terms related to the microbiota-gut-brain axis, intestinal barrier, microbial metabolites, probiotics, prebiotics, synbiotics, postbiotics, psychobiotics and pigs. A representative PubMed search string was: (“gut-brain axis” OR “microbiota-gut-brain axis” OR microbiota OR microbiome OR metabolites) AND (probiotic OR prebiotic*OR synbiotic* OR postbiotic* OR psychobiotic*) AND (intestinal barrier OR gut barrier OR permeability) AND (pig OR pigs OR swine) AND (behavior* OR stress OR neuroendocrine OR “HPA axis”)*. Equivalent terms were adapted for Scopus and Web of Science.

Studies were included if they investigated pigs or provided mechanistic insights relevant to pigs and reported outcomes related to gut microbiota, intestinal barrier function, microbial metabolites, behavioral, or neuroendocrine measures. Reviews, conference abstracts, non-peer-reviewed publications, in vitro-only studies, or studies not available in English were excluded. After screening titles, abstracts and full texts, 92 studies met all eligibility criteria and were included in this review.

## 3. Physiological and Molecular Aspects of Gut-Brain Axis Function

**Figure 1:**
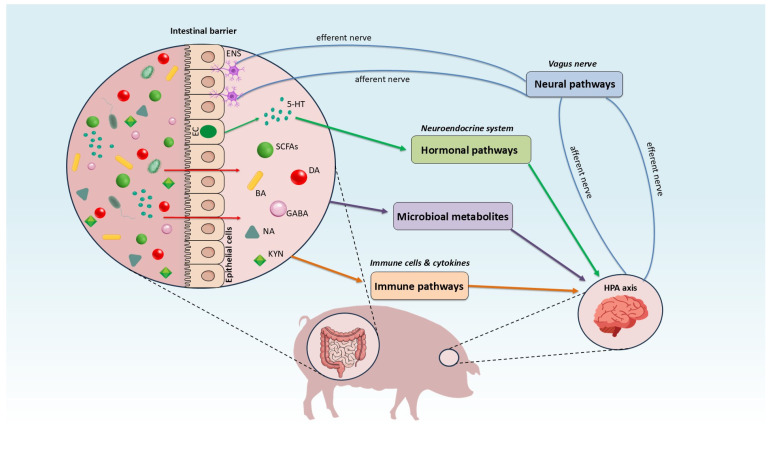
Mechanisms of the gut-brain axis interaction in swine. Blue arrows indicate the neural pathway, originating from the enteric nervous system (ENS), with signals transmitted from the gut to the brain via the vagus nerve. Green arrows represent the hormonal pathway, in which enterochromaffin cells (EC) release serotonin that subsequently signals to the brain. Purple arrows denote gut-to-brain communication mediated by microbiota-derived metabolites. Orange arrows indicate immune-mediated signaling between the gut and the brain. ENS: enteric nervous system, EC: enterochromaffin cells, BA: bile acid, 5-HT: serotonin, GABA: γ-aminobutyric acid, DA: dopamine, NA: norepinephrine, SCFAs: short-chain fatty acids, KYN: tryptophan metabolites (kynurenines).

GBA is a dynamic, bidirectional communication network connecting the digestive tract with the central nervous system (CNS) [15]. This communication occurs primarily through three pathways: the nervous system (mediated by the vagus nerve), the immune system (mediated by immune cells) and the neuroendocrine system (mediated by hormones) [15,16]. The gut microbiota also plays a crucial role, influencing the maintenance of gastrointestinal homeostasis and the integration of the brain’s emotional and cognitive functions with digestive system activity (Figure 1) [15,16,17].

The gut-brain axis is a bidirectional regulatory system connecting the intestines and the central nervous system (CNS) through a variety of pathways. Information from the gut is generated by microbial metabolites (such as neurotransmitters, SCFAs and tryptophan metabolites), which, along with immune cells and cytokines, are transmitted to the brain. This transmission occurs primarily via afferent fibers of the vagus nerve and hormonal and immunological pathways. These metabolites possess the capability to modulate the Hypothalamic-Pituitary-Adrenal (HPA) Axis, thereby influencing stress reactivity and neuroendocrine signaling. Responses from the brain are sent back to the gut through efferent fibers, influencing the function of the intestinal barrier and the activity of the Enteric Nervous System (ENS). This constant signal exchange underpins neuroimmunological regulation, which is essential for maintaining intestinal homeostasis and stable CNS function, directly impacting the organism’s behavior and physiology.

The neural pathway of the gut-brain axis includes the ENS, a network of neurons and glial cells located within the walls of the digestive tract, which plays a critical role in mediating communication between the gut microbiota and the brain. Although it constitutes an integral component of the autonomic nervous system, it is characterized by a high degree of autonomy and the capacity to independently regulate gastrointestinal functions without input from the CNS [18]. The primary communication pathway between the ENS and the brain is the vagus nerve (VN). The VN, the tenth cranial nerve, originates in the brainstem and extends to the proximal portion of the large intestine, innervating numerous organs along its course, including the gastrointestinal tract [19]. The VN receives indirect signals generated by mechanical, chemical and hormonal stimuli. Its afferent sensory endings comprise mechanoreceptors that respond to intestinal wall stretch, chemoreceptors and interoceptive endings within the mucosa. Metabolites produced by the intestinal microbiota, such as short-chain fatty acids, 5-hydroxytryptamine, tryptophan derivatives and bile acids, activate chemosensory afferent neurons of the VN, primarily via enteroendocrine cells (EECs), enabling the gut microbiota to modulate interoceptive signaling. This information is transmitted to the nucleus of the solitary tract (NTS) and subsequently to central nervous system structures. Thus, the VN is considered a key pathway linking the gut-brain axis, regulating emotion recognition and stress responses [19,20,21].

Information regarding the immune status of the intestines is transmitted to the brain via afferent neurons, while efferent signals return through sympathetic or parasympathetic nerves, or directly to the mucosa. Immune cells express receptors for neurotransmitters and neurons respond to cytokines, facilitating bidirectional communication between the nervous and immune systems [22]. Studies in germ-free (GF) animals have demonstrated a reduced number of macrophages, mast cells and T lymphocytes in the intestines, along with decreased IL-22 production, underscoring the role of the microbiota in shaping intestinal immunity [23]. These findings indicate that the microbiota not only supports local intestinal homeostasis but also indirectly modulates gut-brain axis functioning through immune signaling, constituting a key component of neuroimmunological regulation.

EECs, located in the epithelium of the gastrointestinal tract, play a pivotal role in maintaining intestinal homeostasis [24]. In response to nutrients in the intestinal lumen, these cells secrete hormones that modulate local motility and metabolic processes [25]. L cells and enterochromaffin cells are particularly significant in the context of the gut-brain axis. L cells produce peptide YY (PYY) and glucagon-like peptide-1 (GLP-1), which contribute to the neurohormonal regulation of satiety and appetite. Receptors for these peptides are expressed in intestinal neurons, vagus nerve afferent fibers and in central nervous system structures, with their effects on appetite being either direct at the hypothalamic level or indirect via the vagus nerve [24]. Additionally, a communication mechanism has been described in which L cells utilize so-called neuropods—cytoplasmic basolateral projections that form synapses with afferent fibers of the vagus nerve, enabling rapid and precise signal transmission to the central nervous system [26]. The release of GLP-1 and PYY in the proximal intestine occurs primarily in response to nutrients, whereas in the distal intestine their secretion is stimulated by bacterial metabolites, including short-chain fatty acids (SCFAs), bile acids, lipopolysaccharides (LPS) and indole. In this manner, interactions between the gut microbiota and L cells in the distal intestine modulate the activity of these hormones, thereby influencing gut-brain axis function [24].

## 4. Microbiota Regulation of the Intestinal Barrier Integrity and Gut-Brain Axis

The gut microbiota modulates the gut-brain axis primarily through its metabolites, which serve signaling functions. The most important of these include neuroactive compounds such as 5-HT, γ-aminobutyric acid (GABA), dopamine (DA), tryptophan metabolites, SCFAs and secondary bile acids. These substances influence both intestinal barrier integrity and HPA axis activity, thereby modulating inflammatory responses, neurotransmission and immune functions.

In this context, neurotransmitters such as 5-HT, DA, GABA and NA are produced or modulated by specific gut bacterial species. These microorganisms include *Lactobacillus*, *Bifidobacterium* and *Bacteroides*, which can influence the levels of neurotransmitter precursors in the intestinal lumen and synthesize various neurotransmitters, including GABA, 5-HT, DA and NA, that are involved in synaptic regulation and neuronal signal transmission [19,27]. Strains such as *Bifidobacterium*, *Lactobacillus* and *Lactiplantibacillus* are particularly active in GABA production, whereas *Bacillus* and *Escherichia* synthesize dopamine. Although these neurotransmitters do not cross the blood-brain barrier, their levels in the gut remain closely linked to microbiota composition and can modulate physiological functioning, including via the vagus nerve [11]. In animal models, increased GABA availability has been associated with anxiolytic and antidepressant effects, both following oral administration [28] and through strains such as *Lactobacillus brevis* or *Lactobacillus rhamnosus* JB-1, which elevated GABA levels and improved behavioral parameters [29]. Similarly, dopamine synthesized locally in the gut can modulate neuronal activity and contribute to the attenuation of depressive-like behaviors, emphasizing the crucial role of the gut microbiota and its metabolites in modulating neurotransmitters and regulating neurological and behavioral functions.

Serotonin is a key mediator in gut-brain communication. Over 90% of the body’s total serotonin is synthesized by enterochromaffin (EC) cells of the intestinal epithelium [15,30]. This neurotransmitter regulates gastrointestinal motility and participates in signal transmission to the central nervous system, affecting mood, behavior and stress responses, thereby serving as an essential link integrating gut and brain functions. Serotonin synthesis from tryptophan, catalyzed by tryptophan hydroxylase (TPH1), is modulated by gut bacterial metabolites, particularly SCFAs, which enhance TPH1 expression and 5-HT production via GPR41 and GPR43 receptors on EC cells [24,31]. The microbiota also influences serotonin reuptake by modulating the SERT transporter [32]. In weaned piglets, *Lactobacillus amylovorus* increases 5-HT levels in the large intestine, partly through acetate-mediated stimulation of the FFAR3 (GPR41) receptor [33]. Released serotonin acts locally to regulate intestinal motility and secretion and systemically activates 5-HT3 receptors of the vagus nerve, facilitating communication with the central nervous system [15].

In addition to neurotransmitters, metabolites of the gut microbiota, such as SCFAs, tryptophan derivatives and bile acids, play a key role in gut-brain communication. These compounds modulate the expression of tight junction proteins (including ZO-1, occludin, claudins), strengthen the intestinal barrier, regulate the immune response and influence neurotransmission, supporting the integrity of the gut-brain axis [5,6]. Disruptions in their production lead to a weakened intestinal barrier, increased epithelial permeability and activation of the inflammatory response, which in turn can result in the overproduction of lipopolysaccharide (LPS) by Gram-negative bacteria, activation of NADPH oxidase (NOX2) and increased oxidative stress (ROS), exacerbating local inflammation and stimulating the release of pro-inflammatory cytokines (IL-6, TNF-α, IL-1β). These cytokines can enter the bloodstream and affect the CNS, exacerbating neuroinflammatory stress response mechanisms [10,34,35,36].

SCFAs, including acetate, propionate and butyrate, are produced through the anaerobic fermentation of indigestible polysaccharides by intestinal microorganisms in pigs. They play a multifaceted role in maintaining metabolic, immune and neuronal homeostasis. SCFAs modulate microglial maturation, neurotransmitter levels and neurotrophic factors such as BDNF, nerve growth factor (NGF) and glial cell line-derived neurotrophic factor (GDNF), thereby supporting normal neuronal function [8,27]. Concurrently, they exert strong immunomodulatory effects, regulating the activity of Treg cells, T lymphocytes and macrophages in the intestine and attenuating LPS-induced inflammatory responses [37].

Another important aspect of SCFAs activity is their ability to enhance intestinal barrier integrity. SCFAs increase the expression of tight junction proteins (occludin, claudin-1, ZO-1, CLDN4) and reduce nitric oxide secretion. This mechanism is associated with the activation of SCFAs receptors (FFAR2, FFAR3, HCAR2) and transporters MCT1 and SMCT1, whose expression is upregulated in response to these metabolites [38]. Studies in weaned piglets have shown that intragastric infusion of SCFAs increases mRNA levels of occludin and claudin-1, thereby improving intestinal barrier tightness [6]. Similarly, in a co-culture model of intestinal epithelial cells (IPEC-J2) and monocytes (PBMCs), propionate and acetate enhanced the expression of intercellular junction proteins and reduced nitric oxide secretion [39]. In addition, SCFAs influence lipid and glucose metabolism, modulate the composition of the microbiota and the concentration of biogenic amines, which highlights their multifaceted role in regulating gut-brain communication in pigs [40,41].

Secondary bile acids, such as deoxycholic acid and lithocholic acid, represent another important class of metabolites, performing signaling functions via farnesoid X receptors (FXR) and TGR5. Activation of these receptors influences neurotransmitter levels, including acetylcholine, GABA and N-methyl D-aspartate (NMDA), thereby supporting proper gut-brain axis function [12]. Intestinal dysbiosis can impair secondary bile acid synthesis, leading to increased intestinal permeability and disruption of microbial homeostasis [35]. Studies in weaned piglets have demonstrated that supplementation with tauroursodeoxycholic acid (TUDCA) improved intestinal morphology and enhanced tight junction protein expression, suggesting a potential mechanism by which secondary bile acids support both intestinal barrier integrity and brain signaling [42].

Another key element in gut-brain communication is tryptophan metabolism, which primarily follows three pathways: serotonin, kynurenine and indole. The balance among these pathways is critical for proper gut-brain axis function and the maintenance of intestinal barrier integrity. The microbiota regulates tryptophan availability, directing its metabolism toward the synthesis of serotonin or indole and kynurenine metabolites, which exert immunomodulatory or neurotoxic effects [43]. Disruption of this balance due to dysbiosis promotes a shift toward the kynurenine pathway, limiting serotonin production and impairing gut-brain axis function. Pro-inflammatory cytokines and LPS can further activate the enzyme indoleamine 2,3-dioxygenase 1 (IDO1), exacerbating oxidative stress and inflammation, thereby compromising neuroendocrine function and piglet welfare [9,35]. Studies have demonstrated that supplementation with tryptophan at concentrations of 0.2–0.5% or its precursor 5-hydroxytryptophan (5-HTP) at a dose of 250 mg/kg body weight in weaned piglets improves weight gain, feed conversion and reduces the incidence of diarrhea, effects attributed to the modulatory role of serotonin in regulating intestinal motility and alleviating weaning stress [44]. However, excessive supplementation with tryptophan (0.75%) or 5-HTP (500 mg/kg body weight) may disrupt the villus-to-crypt ratio and increase intestinal permeability, likely due to overstimulation of gastrointestinal motility [45].

The integrity of the intestinal barrier is also closely linked to the activity of the HPA axis. Chronic stress leads to increased secretion of glucocorticoids and corticotropin-releasing hormone (CRH), which increases the permeability of the intestinal epithelium and promotes the translocation of LPS and other bacterial toxins [46]. In pigs, especially during weaning, environmental and nutritional stress exacerbates these processes, leading to microbiota disruption, reduced production of beneficial metabolites and decreased growth rate [47]. Thus, gut microbiota metabolites are a key signalling link in the gut-brain axis, connecting intestinal barrier integrity, immune response and neurotransmission in adaptive mechanisms to environmental and production stress.

Proper gut-brain axis function is directly relevant to pig welfare, as it integrates neuroendocrine, immune and metabolic responses under stressful conditions. Disruptions in this communication result in increased HPA axis activity, compromised intestinal barrier integrity and exacerbated stress-related behaviors, such as tail biting, social avoidance and hypervigilance. Consequently, the status of the gut microbiota and its metabolites (SCFAs, 5-HT and bile acids) may serve as key indicators of stress resilience and adaptive capacity in pigs under production conditions.

## 5. Developmental Changes in the Porcine Gut Microbiota and Animal Behavior

Given the key role of the microbiota in regulating gut-brain axis functions, its composition and developmental dynamics are of considerable importance. The porcine gut microbiota undergoes substantial changes during successive growth stages, reaching relative stability only in the finishing period. In the post-weaning phase, its composition is significantly remodeled, representing a transitional stage between the microbiota of suckling piglets and adult pigs. Initially, lactose- and milk oligosaccharide-fermenting bacteria, mainly from the genus *Lactobacillus*, dominate, whereas as the gastrointestinal tract matures, the proportion of *Firmicutes* and *Bacteroidetes*, including *Prevotella*, *Ruminococcus* and *Faecalibacterium*, increases [48]. Weaning is associated with a transient decrease in *Lactobacillus* abundance and an increase in opportunistic bacteria, reflecting dysbiosis and increased susceptibility to pathogen colonization [49]. In light of these changes, an increasing number of reports indicate that the composition and stability of the gut microbiota may be closely linked to neurobehavioral functions in pigs, reflecting gut-brain axis activity (Table 1 and Table 2). Rabhi et al. [7] demonstrated that groups of piglets exhibiting tail-biting behavior had significantly lower *Lactobacillus* abundance and higher serum cortisol levels. Verbeek et al. [50] did not observe a direct association between *Lactobacillus* decline and behavior; however, in tail-biting pigs, increased abundance of *Ruminococcaceae* and *Lachnospiraceae* was noted, which are involved in serotonin biosynthesis and SCFAs production. Despite the increased abundance of these bacteria, SCFAs concentrations in the gut were reduced, suggesting that the relationship between the microbiota, metabolites and behavior is complex and modulated by additional factors such as diet, stress and intestinal absorption.

Choudhury et al. [13] demonstrated that specific bacterial groups, including *Atopobium* and *Prevotella/Prevotellaceae*, were differentially associated with exploratory behavior. *Atopobium* correlated with increased exploratory activity, whereas *Prevotella* correlated with its decrease. Conversely, König et al. [51] reported that piglets exhibiting manipulative behaviors had lower abundances of culturable *Lactobacillaceae* species and reduced microbiota diversity. At the same time, the need for further research was emphasized to clarify the causality of these relationships, as most analyses were observational in nature.

An extremely important aspect of pig husbandry affecting the maintenance of intestinal barrier integrity and proper gut-brain axis communication is the environmental conditions, including housing systems. Stress associated with limited space or lack of environmental stimuli, as in conventional farrowing crates, can lead to gut dysbiosis, increased intestinal epithelial permeability and elevated production of proinflammatory cytokines, which consequently affect neuroendocrine functions and animal behavior [52]. In contrast, environmentally enriched systems that provide more space, access to manipulable materials and opportunities for exploration promote a more favorable microbiota composition, reduce behavioral stress and improve social interactions in pigs [53]. The effects of such microbiota changes may include modulation of SCFAs and neurotransmitter production, which are key mediators of gut-brain axis signaling [6].

In addition to manipulative behaviors, the gut microbiota may also influence other aspects of pig behavior related to stress (Table 1). Studies have shown that gut dysbiosis correlates with reduced appetite, limited exploratory behavior and social withdrawal [54]. A high abundance of *Prevotella* has been associated with apathy and decreased feed intake, whereas the presence of *Lactobacillus reuteri* or *Bifidobacterium animalis* promoted greater exploratory activity and resilience to stressors [7,55]. These observations suggest that the gut microbiota may influence not only aggressive behaviors but also subtle manifestations of stress, which are critical for assessing pig welfare

## 6. The Influence of Nutrition on the Gut Microbiota of Pigs

Nutrition is one of the key factors shaping the gut microbiota of animals. The use of feed additives such as probiotics, prebiotics and synbiotics allows for modulation of the composition and activity of the microbial community, supporting intestinal barrier integrity, gastrointestinal homeostasis and the regulation of immune responses [56,57]. In pigs, their effects are primarily exerted in the cecum and colon, which are inhabited by a rich and diverse microbial population [58].

An increasing number of studies indicate that probiotics influence the functioning of the gut-brain axis, which may contribute to reducing stress responses and improving behavior and welfare in pigs (Table 2). Strains of *Lactobacillus* and *Bifidobacterium* play a significant role in this process by increasing the availability of tryptophan, thereby enhancing the biosynthesis of serotonin and dopamine, which supports mood regulation and cognitive functions. Additionally, these probiotic strains modulate HPA axis activity by lowering cortisol levels and reducing symptoms of anxiety and depression [59]. This is supported by the findings of Pereira et al. [60], who demonstrated that administering to sows during gestation and lactation a mixture of seven probiotic strains (*L. acidophilus*, *L. bulgaricus*, *L. plantarum*, *L. rhamnosus*, *B. bifidum*, *E. faecium and S. thermophilus*) reduced apathy and anxiety, as reflected by a 50% decrease in cortisol levels and an 11% increase in serotonin. Moreover, piglets born to these sows exhibited lower stress levels in behavioral tests.

Similar effects have been observed in piglets receiving *Lactobacillus reuteri* and *Lactobacillus plantarum* during early life. In a study conducted by Verbeek et al. [61], supplementation with *Lactobacillus strains* reduced the intensity of vigilance behaviors (including raising the head to shoulder height or higher) in response to a threatening stimulus. The vigilance level in piglets receiving probiotic supplementation remained similar both before and after exposure to a threatening stimulus, indicating that the probiotic prevented the typical stress response. Consequently, probiotic supplementation effectively blocked the threat-induced increase in vigilance, which can be interpreted as an anxiolytic effect. This suggests that probiotic administration promoted a more appropriate, balanced and less fear-driven response to the stressor in piglets.

*Bifidobacterium animalis*, in turn, positively influences intestinal development, antioxidant capacity and the microbiota of piglets, thereby contributing to improved welfare. Supplementation with *B. animalis* enhances body weight gain and reduces the incidence of diarrhea, stimulates intestinal development by increasing villus height in the duodenum, the number of goblet cells and amylase activity in the jejunum and strengthens the antioxidant capacity of the mucosa while lowering malondialdehyde levels. Microbiome analysis shows an increase in beneficial bacteria (*Streptococcus*, *Erysipelotrichaceae*, *Coprococcus*, *Oscillibacter*) alongside a decrease in pathogenic bacteria (*Helicobacter*, *Escherichia-Shigella*) [62]. *Bacillus amyloliquefaciens* SC06, on the other hand, not only improves intestinal barrier function and antioxidant capacity, thereby positively affecting stress response and gastrointestinal health, but also enhances body weight gain and feed conversion efficiency [63]. Improvements in intestinal structure, microbial balance and antioxidant function with the use of both probiotics contribute to a significant enhancement of piglet welfare.

Modulation of the gut-brain axis is not limited to the effects of probiotics. Prebiotics, through modulation of the gut microbiota, reduction of oxidative stress and attenuation of neuroinflammation, also contribute to the improvement of cognitive functions and overall welfare in piglets. In the study by Tian et al. [64], piglets fed with galactooligosaccharides (GOS) exhibited increased resistance to LPS-induced oxidative stress (reduction of ROS and malondialdehyde (MDA)), higher activity of antioxidant enzymes (GSH-Px—glutathione peroxidase; SOD—superoxide dismutase; T-AOC—total antioxidant capacity; AMPK—AMP-activated protein kinase; HO-1—heme oxygenase-1; NQO1—NAD(P)H quinone oxidoreductase 1) and enhanced expression of tight junction proteins (DAO—diamine oxidase; ZO-1—zonula occludens-1; Claudin-1—tight junction protein claudin-1; Occludin—tight junction protein occludin). Similar effects, including the reduction of proinflammatory cytokines, increased butyrate levels and improved cognitive function, have also been observed in rodent studies, indicating that the antioxidant, anti-inflammatory and neuroprotective mechanisms of prebiotics may operate across different species [65].

The combined action of probiotics and prebiotics in the form of a synbiotic may enhance these effects, as confirmed by experimental studies. Furthermore, in the study by Parois et al. [66], a synbiotic containing three *Lactobacillus* spp. strains, fructooligosaccharides (FOS) and beta-glucan positively influenced cognitive abilities in piglets and modulated gut microbiota composition, increasing the abundance of *Lactobacillus* and *Bifidobacterium* while simultaneously reducing populations of pathogenic microorganisms [66]. This effect was associated with attenuation of neuroinflammation and improvement of cognitive function, indicating a significant involvement of the gut-brain axis in the mechanism of synbiotic action. Concurrently, postbiotics, defined as preparations of non-viable microorganisms or their components, represent another important factor modulating the microbiome and the gut-brain axis. They are characterized by the absence of live microorganisms and may consist of whole inactivated cells, cell fragments (e.g., cell walls), or metabolic products such as SCFAs, enzymes, vitamins, or exopolysaccharides. Their effects include immunomodulation, anti-inflammatory and antioxidant activity and reinforcement of the intestinal barrier [67], allowing the beneficial outcomes achieved with probiotics and prebiotics to be maintained even in the absence of live microorganisms. Studies conducted in pigs have shown that supplementation with tributyrin, a butyrate precursor, improved spatial memory and synaptic plasticity in the hippocampus through activation of PPARγ and AMPK pathways, which support energy homeostasis and neuroprotection [68]. However, research by Sutkus et al. [69] indicated that under conditions of severe inflammatory stress, administration of butyrate alone may not be sufficient to protect the brain and maintain proper gut-brain axis communication, highlighting the need for more complex interventions.

**Table 2:** Effects of feed additives on gut-brain axis, behavior and stress responses in experimental vs. control group.

Production Group	N	Experimental Group	Duration	Results	Behavioral/Stress Effect	Study
Sows (Pic Camborough, Agroceres-PIC)	147	Multi-strain probiotic: *L. acidophilus*, *bulgaricus*, *plantarum*, *rhamnosus* *B. bifidum* *E. faecium* *S. thermophilus*	From insemination to 21 days of piglet age	↑ 5-HT (*p* = 0.034) ↓ Cortisol (*p* = 0.047)	↓ apathy, ↓ anxiety ↓ cortisol in saliva ↑ serotonin in blood	[60]
Duroc piglets × (Landrace × Yorkshire)	35	Synbiotic: 3 *Lactobacillus* strains (*L. salivarius*, *L. reuteri*) + FOS + beta-glucan + vitamin C	Days 1–28 of age	↑*Bacteroidetes* (*p* = 0.017), ↓*Firmicutes* (*p* = 0.012)	↑ interaction speed ↑ problem-solving ability ↑ learning flexibility	[66]
Suckling piglets (Specific pathogen-free sows)	64	Probiotic: *L. reuteri* ATCC-PTA-6475 *L. plantarum* L1-6	Days 3–35 of age		↓ anxiety ↓ increased vigilance ↑ attention to threat	[61]
Suckling piglets (Landrace × Duroc × Yorkshire)	18	Prebiotic: GOS	Days 1–14 of age	↓ ROS (*p* < 0.05) ↓ MDA (*p* < 0.05) ↑ AMPK (*p* < 0.05)	↓ oxidative stress ↑ digestive and absorptive functions	[64]
Male piglets (PIC Line 3 dams, pooled semen source)	24	Postbiotic: TBCD	Days 1–26/27 of age	↓ MWF (*p* = 0.003) ↓ TBV (*p* = 0.006) ↓ GMV (*p* = 0.013) ↓ FA (*p* < 0.05)	↑ feed aversion x lack of health-promoting effects	[69]
Weaning piglets (Duroc × Landrace × Large White)	96	Probiotic: *Bifidobacterium animalis subsp. lactis* JYBR-190	Days 1–28 of age	↑ BW (*p* = 0.022) ↑ ADG (*p* < 0.05) ↑ T-AOC (0.003) ↓ MDA (*p* = 0.037) ↑ Amylase activity (*p* = 0.049) ↓*Helicobacter i Escherichia-Shigella* (*p* < 0.05)	↓diarrhoea ↑ body weight	[62]
Male piglets (Duroc Landrace Yorkshire)	32	Probiotic: *Bacillus amyloliquefaciens* SC06 (SC06)	Days 1–29 of age	↑ ADG (*p* = 0.03) ↓ DAO (*p* < 0.01) ↓ D-lactate (*p* < 0.01) ↓*Clostridium* (*p* < 0.01) ↑*Actinobacillus* (*p* < 0.05)	↑ growth performance ↑ Intestinal barrier health and function ↓ stress	[63]

N—Number of animals; Duration—duration of experiment; ↑ Increase; ↓ Decrease; 5-HT—5-hydroxytryptamine (serotonin); DAODiamine oxidase; GOS—Galacto-oligosaccharides; AMPK—AMP-activated protein kinase; TBCD—Tributyrin encapsulated with gamma-cyclodextrin; FOS—fructooligosaccharide; ROS—Reactive oxygen species; MDA—Malondialdehyde; AMPK—AMP-activated protein kinase; MWF—Myelin Water Fraction; FA—Fractional anisotropy (an indicator of fiber organization and white matter microstructure); TBV—total brain volume; GMV—gray matter volume; BW—Body weight; ADG -Average daily gain; T-AOC—Total antioxidant capacity.

## 7. The Importance of the Gut-Brain Axis in Modern Pig Production

The proper functioning of the gut-brain axis is an increasingly common subject of research in pig breeding, as it integrates metabolic, immunological and neuroendocrine signals, with the gut microbiota playing a key regulatory role in the system. The composition and activity of the microbiota affect not only the animals’ immunity and response to infections, but also behaviors related to stress and aggression [70].

The intestinal microbiota of pigs is strongly dependent on diet and changes in feeding practices at different stages of production lead to significant modifications in its composition. This is particularly evident during periods critical to health and welfare, such as weaning, when sudden dietary changes, separation from the sow and environmental stress promote intestinal dysbiosis in piglets, leading to frequent diarrhea, increased risk of postweaning diarrhea (PWD) and decreased growth rates [71]. Traditionally, in breeding practice, this problem has been mitigated by the use of zinc oxide and therapeutic antibiotics. However, their use in practice is increasingly restricted due to environmental risks and growing microbial resistance [72,73]. This has led to growing interest in feed additives that modulate the intestinal microbiota, such as probiotics, prebiotics, synbiotics and others, which not only improve intestinal health and reduce the incidence of diarrhea, but also reduce stress and aggression, including the risk of cannibalism and consequently have a positive effect on animal welfare and production efficiency. Supporting the intestinal microflora with such additives translates into improved weight gain and feed conversion. A study by Méndez-Palacios et al. [74] demonstrated that supplementation from weaning to fattening with 4 kg/ton of prebiotic and 0.8 kg/ton of probiotic increased weight gain and improved feed conversion. Economic analysis revealed a marginal return on investment of 7.5:1, demonstrating that modulation of the intestinal microflora is a sustainable and practical tool in modern pig farming. Stabilization of the microbiota promotes the regulation of serotonin, a key neurotransmitter that determines stress responses and social behavior, which in turn improves relationships within the herd [75]. At the same time, the way sows are fed indirectly affects production results by modifying the intestinal microbiota of their offspring. Studies on Yorkshire sows have shown that feeding using an automatic feeding station (SM) increased the number of beneficial bacteria and the concentration of bacterial metabolites in feces, including short-chain acids: butyric, isovaleric, valeric and isobutyric. These changes in microbiota resulted in a higher number of piglets born and a lower number of dead or deformed piglets [76]. Similar mechanisms are observed in sows during pregnancy and lactation, when hormonal and metabolic changes modify the composition of the microbiota, which affects appetite regulation, stress adaptation and nutrient utilization efficiency. Probiotic supplementation stabilizes the microflora, supports the control of neuroendocrine responses and mitigates stress associated with pregnancy and lactation. At the same time, bacterial metabolites transferred to piglets in utero and through milk play an important role in shaping the development of the gut-brain axis and immunity in young animals [77]. In this way, modulation of the intestinal microbiota is a key element of the strategy to improve health, welfare and production performance in modern pig farming, integrating nutritional, immunological and neuroendocrine aspects into a coherent regulatory mechanism. In the case of fattening pigs, transport and pre-slaughter stress remain a particularly significant problem, reducing animal welfare and negatively affecting meat quality. Accelerated muscle glycogenolysis and a decrease in the meat’s ability to retain water are the most common consequences [78].

A growing number of studies indicate that feed additives stabilizing the intestinal microbiota can reduce transport and pre-slaughter stress, improving both animal behavior and meat quality characteristics. At the same time, reducing aggression in the herd reduces the risk of injury and cannibalism, which translates into better zoohygienic conditions and overall welfare [21,79].

Supplementing feed with additives that modulate the intestinal microbiota (e.g., probiotics, prebiotics) brings both measurable health and economic benefits. Analyses indicate that in weaned piglets, the use of *Lactobacillus* and *Bacillus* strains improves feed conversion by 3–7% and reduces mortality by 10–15%, with a return on investment (ROI) of 5–8:1 and a slight increase in feed costs of 2–3% [80]. These benefits are primarily due to the modulation of the intestinal microbiota, which increases the synthesis of 5-HT and SCFAs, improves the integrity of the intestinal epithelium and reduces the inflammatory response. As a result, pigs show reduced stress susceptibility (lower cortisol levels), higher feed intake, enhanced growth rates and carcass quality, which together translate into improved welfare and production efficiency. Industry experience in several EU countries demonstrates that strategies based on microbiota modulation can be implemented in practice, confirming both the biological and economic benefits of using probiotics and prebiotics in pig nutrition.

## 8. Challenges and Directions for Future Research

Research on the intestinal microbiota of pigs clearly confirms its key role in maintaining physiological and immune homeostasis and in regulating stress responses and social behavior. In recent years, increasing attention has been paid to its role in the functioning of the gut-brain axis, which is a multidirectional communication system between the gastrointestinal tract, the central nervous system and the immune system. In the coming years, it will be particularly important to develop probiotic and nutritional programs tailored to the health status of the herd, the age of the animals and environmental conditions, which can support the functioning of the gut-brain axis by stabilizing the microbiota, increasing the production of neuroactive metabolites and improving the integrity of the intestinal barrier [81]. The use of probiotic strains with psychobiotic properties, i.e., the ability to synthesize or modulate neurotransmitters (including 5-HT, DA and GABA), opens up new possibilities for alleviating weaning stress and improving welfare of pigs.

Biotechnological research is also developing rapidly, including the selection of strains with neuroendocrine properties and the engineering of microorganisms capable of synthesizing neurotransmitters such as serotonin and GABA. In pigs, attempts are already being made to develop functional feeds enriched with prebiotics and postbiotics that modulate the activity of intestinal neurons and SCFAs receptors (FFAR2, FFAR3, HCAR2), thereby supporting communication along the gut-brain axis. These approaches offer the possibility of more precise control of stress responses, which in turn can improve welfare, increase animal resistance and reduce the need for high doses of zinc oxide and antibiotics for therapeutic purposes [82]. However, implementing such solutions poses numerous challenges. Key challenges include the difficulty of translating findings from other species into practical pig production systems, the lack of standardization of experimental protocols investigating the gut-brain axis and the limited number of field studies combining the analysis of microbiota, metabolites and animal behavior. Methods for assessing the links between biochemical parameters (e.g., SCFAs, tryptophan, cytokine levels) and stress responses and production performance in pigs are still underdeveloped. Randomized controlled trials involving the use of specific probiotic strains and feed additives are needed to identify the specific mechanisms by which the microbiome influences the central nervous system and animal behavior under production conditions. In addition, the European Union’s stringent legal requirements for the safety and efficacy of feed additives, including probiotics and engineered microorganisms, require multi-stage documentation and costly studies on the target species [14]. Therefore, future research directions should also include the development of new methods for evaluating the effectiveness of microbiome interventions, including biomarkers of stress and intestinal barrier integrity, which could serve as tools for monitoring the impact of diet on the functioning of the gut-brain axis in pigs.

## 9. Multi-Omics Approaches to Elucidating Microbiota-Brain Interactions in Swine

The application of multi-omics technologies has increasingly been recognized as essential for resolving the complex, multilayered interactions that constitute the microbiota-gut-brain axis in pigs. Single-layer analyses as taxonomic profiling alone, provide limited insight into functional activity and host–microbe crosstalk [83]. For this reason, combining complementary omics layers (metagenomics, metatranscriptomics, metabolomics, proteomics and host omics) allows for a complex, systems-level reconstruction of pathways linking microbial taxa to bioactive molecules, intestinal barrier status and neuroendocrine responses [84]. Metagenomic sequencing has been employed to define the taxonomic composition and functional potential of the porcine gut microbiome, enabling the identification of microbial genes and pathways that may encode enzymes for production or transformation of neuroactive compounds [85]. Metagenome-assembled genomes (MAGs) and gene catalogs specific to swine have improved resolution at the strain and gene level, which is critical for distinguishing taxa with differing neurochemical capacities [86,87]. Through these approaches, candidate microbial taxa and gene families associated with performance, health and stress resilience can be prioritized for further functional validation. Moreover, metatranscriptomics complements metagenomics by measuring gene expression in situ of the microbial community and thus revealing which functional pathways are actively transcribed under defined physiological or stress conditions [88]. By capturing microbial activity dynamic changes, metatranscriptomic data permit temporal linkage between environmental or nutritional perturbations and shifts in microbial functions that could modulate intestinal permeability, mucosal immunity, or production of neuroactive metabolites. Such transcriptional evidence strengthens causal inference beyond correlations inferred from DNA-based surveys [84]. In turn, metabolomics provides a direct readout of small molecules that mediate gut-brain communication. Targeted and untargeted metabolomic profiling of feces, digesta, plasma and cerebrospinal fluid has been used to link dietary interventions and microbiome alterations to changes in metabolite pools that are known modulators of intestinal barrier integrity and HPA axis activity. Metabolomic signatures have therefore been proposed as proximate biomarkers of intestinal health and stress reactivity in pig production systems [86].

Integrative multi-omics permits the reconstruction of mechanistic pathways linking microbial taxa to expressed genes, metabolite production, host molecular responses and ultimately behavioral or physiological outcomes. When applied to production-relevant challenges (e.g., weaning stress), integrated datasets have revealed coordinated shifts in microbial composition, active metabolic pathways and host inflammatory or barrier-related gene expression, thereby identifying candidate causal routes for intervention. Such integration additionally facilitates the selection of nutrition-based strategies targeting keystone taxa or functional pathways involved in the modulation of gut permeability and stress-related behaviors [89]. From a practical perspective, multi-omics outputs can be translated into actionable tools for nutrition and welfare management. Biomarkers discovered by metabolomics can be implemented as non-invasive indicators of barrier function or stress. Genomic and transcriptomic signatures of microbial strains with desirable functional traits can inform selection or rational design of probiotic strains and synbiotic formulations [90,91].

Despite existing limitations, the potential of microbiome interventions in pig farming remains significant. Targeted modulation of the gut microbiota can support not only animal health and welfare, but also serve as an effective alternative to antibiotic therapy and practices that increase environmental pressure. A growing body of evidence indicates that the microbiome, through its metabolites, plays an important role in the functioning of the gut-brain axis, influencing immunity, behavior and stress responses in pigs. However, despite the growing number of studies, knowledge of the detailed mechanisms of this communication, especially the role of neurotransmitters in the regulation of intestinal neurotransmission, remains fragmentary. Most of the available results come from short-term or model studies, which makes it difficult to translate them into commercial production conditions. Therefore, future work should focus on long-term field studies using advanced omics techniques (metagenomics, metabolomics, proteomics) to gain a more complete understanding of the microbiota-brain relationship and its links to nutrition and pig welfare. This approach could form the basis for the development of integrated, ethical and sustainable breeding strategies that support the innovative and responsible development of the pig sector.

## 10. Summary

The pig gut microbiota plays an important role in the gut-brain axis, influencing neuroendocrine, immune and metabolic functions. Maintaining microbial balance may contribute to the integrity of the intestinal barrier and modulate responses to environmental stress. Experimental and associative studies suggest that microbiota-targeted interventions, such as probiotics, prebiotics, or stage-specific feeding strategies, have the potential to affect behavior and physiological responses, although evidence from commercial production settings remains limited. Future research should focus on elucidating the mechanisms underlying interactions between the microbiota, neurohormonal axes and pig behavior, as well as assessing the practical applicability and economic feasibility of such interventions. An integrated approach combining microbiology, neurophysiology and production management may help inform sustainable strategies to support pig health and welfare.

## Figures and Tables

**Table 1:** Relationship between gut microbiota composition and behavioral traits in pigs.

Production Group	N	Duration	Behavioral Group		Microbiota Changes		Study
Finishing pigs (Landrace × Large White hybrid sows sired with Duroc × synthetic hybrid boar)	352	4 weeks	Tail-biting pigs (aggressor)/bitten finisher pigs (victim)	Aggressor	↑*Coprococcus* ↑*Clostridium IV*	↓*Lactobacillus*	[7]
				Victim	↑*Sphaerochaeta* ↑*Blautia*, ↑*Phascolarctobacterium*	↓*Lactobacillus*	
Swine (Swedish Landrace Yorkshire × Hampshire)	29	2 years	Tail-biting piglets (aggressor)/bitten piglets (victim)	Aggressor	↑*Firmicutes* (mainly *Lachnospiraceae*, *Ruminococcaceae*)		[50]
				Victim	↑*Firmicutes* (mainly *LachnospiraceaeiButyrivibrio*), ↑ *Bacteroidetes* (*Alloprevotella*)	↓*Bacteroidetes* (mainly ↓*Prevotella 7*), ↓*Proteobacteria* (*Ralstonia*)	
Piglets (Finnish Landrace × Yorkshire × Duroc)	30	Single-day study (day 45)	Piglets exhibiting manipulative behavior (manipulators)	Manipulators	↑ *Lactobacillus amylovorus*	↓ diversity of *Lactobacillaceae*	[51]
Suckling piglets (2-4 tyg.) Topigs-20	47	4 weeks	Piglets exhibiting increased/decreased exploratory activity	↑ Exploratory activity	↑*Atopobium* ↑*Coprococcus 3* ↑*CAG-873* ↑*Eubacterium* ↑*Coprostanoligenes*		[13]
				↓ Exploratory activity	↑*Prevotella 9*		

N—number of animals; Duration—duration of experiment; ↑ Increase; ↓ Decrease.

## Data Availability

Not applicable—this article is a review paper and does not report any new data.

## References

[1] Kraimi N., Dawkins M., Gebhardt-Henrich S.G., Velge P., Rychlik I., Volf J., Creach P., Smith A., Colles F., Leterrier C. (2019). Influence of the Microbiota-Gut-Brain Axis on Behavior and Welfare in Farm Animals: A Review. Physiol. Behav..

[2] Niu X., Ding Y., Chen S., Gooneratne R., Ju X. (2022). Effect of Immune Stress on Growth Performance and Immune Functions of Livestock: Mechanisms and Prevention. Animals.

[3] Carabotti M., Scirocco A., Maselli M.A., Severi C. (2015). The Gut-Brain Axis: Interactions between Enteric Microbiota, Central and Enteric Nervous Systems. Ann. Gastroenterol..

[4] Arneth B. (2025). Gut-Brain Axis and Brain Microbiome Interactions from a Medical Perspective. Brain Sci..

[5] Lo B.C., Chen G.Y., Núñez G., Caruso R. (2021). Gut microbiota and systemic immunity in health and disease. Int. Immunol..

[6] Diao H., Jiao A.R., Yu B., Mao X.B., Chen D.W. (2019). Gastric infusion of short-chain fatty acids can improve intestinal barrier function in weaned piglets. Genes Nutr..

[7] Rabhi N., Dicksved J., Keeling L., Landberg R. (2020). Association between tail-biting and intestinal microbiota composition in pigs. Front. Vet. Sci..

[8] Parker A., Fonseca S., Carding S.R. (2020). Gut microbes and metabolites as modulators of blood-brain barrier integrity and brain health. Gut Microbes.

[9] Hestad K., Alexander J., Rootwelt H., Aaseth J.O. (2022). The role of tryptophan dysmetabolism and quinolinic acid in depressive and neurodegenerative diseases. Biomolecules.

[10] Kearns R. (2024). Gut-Brain Axis and Neuroinflammation: The Role of Gut Permeability and the Kynurenine Pathway in Neuro-logical Disorders. Cell. Mol. Neurobiol..

[11] Jiang C., Chen Y., Sun T. (2025). From the gut to the brain, mechanisms and clinical applications of γ-aminobutyric acid (GABA) on the treatment of anxiety and insomnia. Front. Neurosci..

[12] Kiriyama Y., Nochi H. (2019). The Biosynthesis, Signaling, and Neurological Functions of Bile Acids. Biomolecules.

[13] Choudhury R., Middelkoop A., Bolhuis J.E., Kleerebezem M. (2022). Exploring the Association between Microbiota and Behaviour in Suckling Piglets. Sci. Rep..

[14] Chen S., Luo S., Yan C. (2021). Gut Microbiota Implications for Health and Welfare in Farm Animals: A Review. Animals.

[15] Hwang Y.K., Oh J.S. (2025). Interaction of the Vagus Nerve and Serotonin in the Gut-Brain Axis. Int. J. Mol. Sci..

[16] Lu S., Zhao Q., Guan Y., Sun Z., Li W., Guo S., Zhang A. (2024). The communication mechanism of the gut-brain axis and its effect on central nervous system diseases: A systematic review. Biomed. Pharmacother..

[17] Wachsmuth H.R., Weninger S.N., Duca F.A. (2022). Role of the gut–brain axis in energy and glucose metabolism. Exp. Mol. Med..

[18] Spencer N.J., Hu H. (2020). Enteric nervous system: Sensory transduction, neural circuits and gastrointestinal motility. Nat. Rev. Gastroenterol. Hepatol..

[19] Kim Y., Lim J., Oh J. (2024). Taming neuroinflammation in Alzheimer’s disease: The protective role of phytochemicals through the gut-brain axis. Biomed. Pharmacother..

[20] Jameson K.G., Kazmi S.A., Ohara T.E., Son C., Yu K.B., Mazdeyasnan D., Leshan E., Vuong H.E., Paramo J., Lopez-Romero A. (2025). Select microbial metabolites in the small intestinal lumen regulate vagal activity via receptor-mediated signaling. iScience.

[21] Wang L., Wang C., Peng Y., Zhang Y., Liu Y., Liu Y., Yin Y. (2023). Research progress on anti-stress nutrition strategies in swine. Anim. Nutr..

[22] Fukasawa N., Tsunoda J., Sunaga S., Kiyohara H., Nakamoto N., Teratani T., Mikami Y., Kanai T. (2025). The gut-organ axis: Clinical aspects and immune mechanisms. Allergol. Int..

[23] Delgado-Ocaña S., Cuesta S. (2024). From microbes to mind: Germ-free models in neuropsychiatric research. mBio.

[24] Cryan J.F., O’Riordan K.J., Cowan C.S.M., Sandhu K.V., Bastiaanssen T.F.S., Boehme M., Codagnone M.G., Cussotto S., Fulling C., Golubeva A.V. (2019). The Microbiota-Gut-Brain Axis. Physiol. Rev..

[25] Gribble F.M., Reimann F. (2016). Enteroendocrine Cells: Chemosensors in the Intestinal Epithelium. Annu. Rev. Physiol..

[26] Faraji N., Payami B., Ebadpour N., Gorji A. (2025). Vagus nerve stimulation and gut microbiota interactions: A novel therapeutic avenue for neuropsychiatric disorders. Neurosci. Biobehav. Rev..

[27] Strandwitz P. (2018). Neurotransmitter modulation by the gut microbiota. Brain Res..

[28] He Y., Ouyang J., Hu Z., Yang J., Chu Y., Huang S., Yang Y., Liu C. (2019). Intervention mechanism of repeated oral GABA administration on anxiety-like behaviors induced by emotional stress in rats. Psychiatry Res..

[29] Braga J.D., Thongngam M., Kumrungsee T. (2024). Gamma-aminobutyric acid as a potential postbiotic mediator in the gut-brain axis. npj Sci. Food.

[30] Choi W., Moon J.H., Kim H. (2020). Serotonergic regulation of energy metabolism in peripheral tissues. J. Endocrinol..

[31] Buey B., Forcén A., Grasa L., Layunta E., Mesonero J.E., Latorre E. (2023). Gut Microbiota-Derived Short-Chain Fatty Acids: Novel Regulators of Intestinal Serotonin Transporter. Life.

[32] Yaghoubfar R., Behrouzi A., Fateh A., Nojoumi S.A., Vaziri F., Khatami S., Siadat S.D. (2021). Effects of *Akkermansiamuciniphila* and *Faecalibacteriumprausnitzii* on serotonin transporter expression in intestinal epithelial cells. J. Diabetes Metab. Disord..

[33] Liu B., Yu D., Sun J., Wu X., Xin Z., Deng B., Fan L., Fu J., Ge L., Ren W. (2022). Characterizing the influence of gut microbiota on host tryptophan metabolism with germ-free pigs. Anim. Nutr..

[34] Reyes-Martínez S., Segura-Real L., Gómez-García A.P., Tesoro-Cruz E., Constantino-Jonapa L.A., Amedei A., Aguirre-García M.M. (2023). Neuroinflammation, Microbiota-Gut-Brain Axis, and Depression: The Vicious Circle. J. Integr. Neurosci..

[35] Silva D.F., Empadinhas N., Cardoso S.M., Esteves A.R. (2022). Neurodegenerative Microbially-Shaped Diseases: Oxidative Stress Meets Neuroinflammation. Antioxidants.

[36] Feng Y., Huang Y., Wang Y., Wang P., Song H., Wang F. (2019). Antibiotics induced intestinal tight junction barrier dysfunction is associated with microbiota dysbiosis, activated NLRP3 inflammasome and autophagy. PLoS ONE.

[37] Rosser E.C., Piper C.J.M., Matei D.E., Blair P.A., Rendeiro A.F., Orford M., Alber D.G., Krausgruber T., Catalan D., Klein N. (2020). Microbiota-derived metabolites suppress arthritis by amplifying aryl-hydrocarbon receptor activation in regulatory B cells. Cell Metab..

[38] Metzler-Zebeli B.U., Koger S., Sharma S., Sener-Aydemir A., Ruczizka U., Kreutzmann H., Ladinig A. (2022). Short-Chain Fatty Acids Modulate Permeability, Motility and Gene Expression in the Porcine Fetal Jejunum Ex Vivo. Nutrients.

[39] Andrani M., Borghetti P., Ravanetti F., Cavalli V., Ferrari L., De Angelis E., Martelli P., Saleri R. (2023). Acetate and propionate effects in response to LPS in a porcine intestinal co-culture model. Porc. Health Manag..

[40] Zhou H., Yu B., Sun J., Chen D., Chen H., Mao X., Huang Z., Zheng P., Yu J., Luo Y. (2021). Short-Chain Fatty Acids Can Improve Lipid and Glucose Metabolism Independently of the Pig Gut Microbiota. J. Anim. Sci. Biotechnol..

[41] Qi R., Qiu X., Du L., Wang J., Wang Q., Huang J., Liu Z. (2021). Changes of Gut Microbiota and Its Correlation with Short-Chain Fatty Acids and Bioamines in Piglets at the Early Growth Stage. Front. Vet. Sci..

[42] Song M., Zhang F., Fu Y., Yi X., Feng S., Liu Z., Deng D., Yang Q., Yu M., Zhu C. (2022). Tauroursodeoxycholic Acid (TUDCA) Improves Intestinal Barrier Function Associated with the TGR5-MLCK Pathway and the Alteration of Serum Metabolites and Gut Bacteria in Weaned Piglets. J. Anim. Sci. Biotechnol..

[43] Hou Y., Li J., Ying S. (2023). Tryptophan Metabolism and Gut Microbiota: A Novel Regulatory Axis Integrating the Microbiome, Immunity, and Cancer. Metabolites.

[44] Xia Y., Peng X., Mao J., Li Z., Zhang H., Wang Q. (2024). Dietary 5-Hydroxytryptophan Supplementation Improves Growth Performance and Intestinal Health of Weaned Piglets. Porc. Health Manag..

[45] Tossou M.C., Liu H., Bai M., Chen S., Cai Y., Duraipandiyan V., Liu H., Adebowale T.O., Al-Dhabi N.A., Long L. (2016). Effect of High Dietary Tryptophan on Intestinal Morphology and Tight Junction Protein of Weaned Pig. BioMed Res. Int..

[46] Sharan P., Vellapandian C. (2024). Hypothalamic-Pituitary-Adrenal (HPA) Axis: Unveiling the Potential Mechanisms Involved in Stress-Induced Alzheimer’s Disease and Depression. Cureus.

[47] Missiego-Beltrán J., Beltrán-Velasco A.I. (2024). The role of microbial metabolites in the progression of neurodegenerative diseases-Therapeutic approaches: A comprehensive review. Int. J. Mol. Sci..

[48] Luo Y., Ren W., Smidt H., Wright A.G., Yu B., Schyns G., McCormack U.M., Cowieson A.J., Yu J., He J. (2022). Dynamic Distribution of Gut Microbiota in Pigs at Different Growth Stages: Composition and Contribution. Microbiol. Spectr..

[49] Shin D., Chang S.Y., Bogere P., Won K., Choi J.Y., Choi Y.J., Lee H.K., Hur J., Park B.Y., Kim Y. (2019). Beneficial Roles of Probiotics on the Modulation of Gut Microbiota and Immune Response in Pigs. PLoS ONE.

[50] Verbeek E., Keeling L., Landberg R., Lindberg J.E., Dicksved J. (2021). The Gut Microbiota and Microbial Metabolites Are Associated with Tail Biting in Pigs. Sci. Rep..

[51] König E., Heponiemi P., Kivinen S., Räkköläinen J., Beasley S., Borman T., Collado M.C., Hukkinen V., Junnila J., Lahti L. (2024). Fewer Culturable *Lactobacillaceae* Species Identified in Faecal Samples of Pigs Performing Manipulative Behaviour. Sci. Rep..

[52] Everaert N., Van Cruchten S., Weström B., Bailey M., Van Ginneken C., Thymann T., Pieper R. (2017). A Review on Early Gut Maturation and Colonization in Pigs, Including Biological and Dietary Factors Affecting Gut Homeostasis. Anim. Feed Sci. Technol..

[53] Wen C., van Dixhoorn I., Schokker D., Smidt H., de Groot J., Kaal-Lansbergen L.M.T.E., Reimert I., van der Meer I.M., van Nes A., van der Waaij L.A. (2021). Environmentally Enriched Housing Conditions Affect Pig Welfare, Immune System and Gut Microbiota in Early Life. Anim. Microbiome.

[54] Yang H., Yang M., Fang S., Huang X., He M., Ke S., Huang L. (2018). Evaluating the Profound Effect of Gut Microbiome on Host Appetite in Pigs. BMC Microbiol..

[55] Amat S., Lantz H., Munyaka P.M., Willing B.P. (2020). Prevotella in Pigs: The Positive and Negative Associations with Production and Health. Microorganisms.

[56] Liao S.F., Nyachoti M. (2017). Using Probiotics to Improve Swine Gut Health and Nutrient Utilization. Anim. Nutr..

[57] Plaza-Diaz J., Ruiz-Ojeda F.J., Gil-Campos M., Gil A. (2019). Mechanisms of Action of Probiotics. Adv. Nutr..

[58] Rossi R., Mainardi E. (2025). Prebiotics and Probiotics Supplementation in Pigs as a Model for Human Gut Health and Disease. Biomolecules.

[59] Rathore K., Shukla N., Naik S., Sambhav K., Dange K., Bhuyan D., Imranul Haq Q.M. (2025). The Bidirectional Relationship Between the Gut Microbiome and Mental Health: A Comprehensive Review. Cureus.

[60] Pereira M.M.C., Andretta I., Franceschi C.H., Kipper M., Mariani A., Stefanello T., Carvalho C., Vieira J., Moura Rocha L., Ribeiro A.M.L. (2024). Effects of Multistrain Probiotic Supplementation on Sows’ Emotional and Cognitive States and Progeny Welfare. Animals.

[61] Verbeek E., Dicksved J., Keeling L. (2021). Supplementation of Lactobacillus Early in Life Alters Attention Bias to Threat in Piglets. Sci. Rep..

[62] Pang J., Liu Y., Kang L., Ye H., Zang J., Wang J., Han D. (2022). Bifidobacterium animalis Promotes the Growth of Weaning Piglets by Improving Intestinal Development, Enhancing Antioxidant Capacity, and Modulating Gut Microbiota. Appl. Environ. Microbiol..

[63] Yuan J., Meng H., Liu Y., Wang L., Zhu Q., Wang Z., Liu H., Zhang K., Zhao J., Li W. (2024). Bacillus amyloliquefaciens Attenuates the Intestinal Permeability, Oxidative Stress and Endoplasmic Reticulum Stress: Transcriptome and Microbiome Analyses in Weaned Piglets. Front. Microbiol..

[64] Tian S., Wang J., Gao R., Wang J., Zhu W. (2022). Early-Life Galacto-Oligosaccharides Supplementation Alleviates the Small Intestinal Oxidative Stress and Dysfunction of Lipopolysaccharide-Challenged Suckling Piglets. J. Anim. Sci. Biotechnol..

[65] Vijaya A.K., Kuras S., Šimoliūnas E., Mingaila J., Makovskytė K., Buišas R., Daliri E.B., Meškys R., Baltriukienė D., Burokas A. (2024). Prebiotics Mitigate the Detrimental Effects of High-Fat Diet on Memory, Anxiety and Microglia Functionality in Ageing Mice. Brain Behav. Imm..

[66] Parois S.P., Eicher S.D., Lindemann S.R., Marchant J.N. (2021). Potential Improvements of the Cognition of Piglets through a Synbiotic Supplementation from 1 to 28 Days via the Gut Microbiota. Sci. Rep..

[67] Vinderola G., Sanders M.E., Salminen S. (2022). The Concept of Postbiotics. Foods.

[68] Sanz-Martos A.B., Fernández-Felipe J., Merino B., Cano V., Ruiz-Gayo M., Del Olmo N. (2022). Butyric Acid Precursor Tributyrin Modulates Hippocampal Synaptic Plasticity and Prevents Spatial Memory Deficits: Role of PPARγ and AMPK. Int. J. Neuropsychopharmacol..

[69] Sutkus L.T., Sommer K.M., Li Z., Sutton B.P., Donovan S.M., Dilger R.N. (2025). Correction: Experimentally Induced Colitis Impacts Myelin Development and Home-Cage Behavior in Young Pigs Regardless of Supplementation with Oral Gamma-Cyclodextrin-Encapsulated Tributyrin. Front. Neurosci..

[70] Lyte J.M., Lyte M. (2019). Review: Microbial Endocrinology: Intersection of Microbiology and Neurobiology Matters to Swine Health from Infection to Behavior. Animals.

[71] Connolly K.R., Sweeney T., P’Doherty J.V. (2025). Sustainable nutritional strategies for gut health in weaned pigs: The role of reduced dietary crude protein, organic acids and butyrate production. Animals.

[72] Su W., Li Z., Gong T., Wang F., Jin M., Wang Y., Lu Z. (2023). An Alternative ZnO with Large Specific Surface Area: Preparation, Physicochemical Characterization and Effects on Growth Performance, Diarrhea, Zinc Metabolism and Gut Barrier Function of Weaning Piglets. Sci. Total Environ..

[73] Tag X., Xiong K., Zeng Y., Fang R. (2024). The Mechanism of Zinc Oxide in Alleviating Diarrhea in Piglets after Weaning: A Review from the Perspective of Intestinal Barrier Function. Int. J. Mol. Sci..

[74] Méndez-Palacios N., Méndez-Mendoza M., Vázquez-Flores F., Castro-Colombres J.G., Ramírez-Bribiesca J.E. (2018). Productive and Economic Parameters of Pigs Supplemented from Weaning to Finishing with Prebiotic and Probiotic Feed Additives. Anim. Sci. J..

[75] Shen C., Tong X., Chen R., Gao S., Liu X., Schinckel A.P., Li Y., Xu F., Zhou B. (2020). Identifying Blood-Based Biomarkers Associated with Aggression in Weaned Pigs after Mixing. Appl. Anim. Behav. Sci..

[76] Wang M., Yue J., Lv G., Wang Y., Guo A., Liu Z., Yu T., Yang G. (2024). Effects of Interactions between Feeding Patterns and the Gut Microbiota on Pig Reproductive Performance. Animals.

[77] Du H., Li K., Guo W., Na M., Zhang J., Zhang J., Na R. (2025). Physiological and Microbial Community Dynamics in Does During Mid-Gestation to Lactation and Their Impact on the Growth, Immune Function, and Microbiome Transmission of Offspring Kids. Animals.

[78] Potes Y., Oliván M., Rubio-González A., de Luxán-Delgado B., Díaz F., Sierra V., Arroyo L., Peña R., Bassols A., González J. (2017). Pig Cognitive Bias Affects the Conversion of Muscle into Meat by Antioxidant and Autophagy Mechanisms. Animal.

[79] Lee I.K., Kye Y.C., Kim G., Kim H.W., Gu M.J., Umboh J., Maaruf K., Kim S.W., Yun C.H. (2016). Stress, Nutrition, and Intestinal Immune Responses in Pigs-A Review. Asian-Australas.J. Anim. Sci..

[80] Yu J., Zuo B., Li Q., Zhao F., Wang J., Huang W., Sun Z., Chen Y. (2024). Dietary Supplementation with *Lactiplantibacillus plantarum* P-8 Improves the Growth Performance and Gut Microbiota of Weaned Piglets. Microbiol. Spectr..

[81] Wang J., Tong T., Yu C., Wu Q. (2025). The Research Progress on the Impact of Pig Gut Microbiota on Health and Production Performance. Front. Vet. Sci..

[82] Li B., Li M., Luo Y., Li R., Li W., Liu Z. (2022). Engineered 5-HT producing gut probiotic improves gastrointestinal motility and behavior disorder. Front. Cell. Infect. Microbiol..

[83] Su F., Su M., Wei W., Wu J., Chen L., Sun X., Liu M., Sun S., Mao R., Bourgonje A.R. (2025). Integrating Multi-Omics Data to Reveal the Host–Microbiota Interactome in Inflammatory Bowel Disease. Gut Microbes.

[84] Xu J., Xu R., Jia M., Su Y., Zhu W. (2021). MetatranscriptomicAnalysis of Colonic Microbiota’s Functional Response to Different Dietary Fibers in Growing Pigs. Anim. Microbiome.

[85] Liu S., Feng B., Zhang Z., Miao J., Lai X., Zhao W., Xie Q., Ye X., Cao C., Yu P. (2025). UPGG: Expanding the Taxonomic and Functional Diversity of the Pig Gut Microbiome with an Enhanced Genome Catalog. NPJ Biofilms Microbiomes.

[86] Chen L.H., Canibe N., Curtasu M.V., Hedemann M.S. (2025). Untargeted Metabolomics as a Tool to Assess the Impact of Dietary Approaches on Pig Gut Health: A Review. J. Anim. Sci. Biotechnol..

[87] Yang B., Yang J., Chen R., Chai J., Wei X., Zhao J., Zhao Y., Deng F., Li Y. (2024). Metagenome-Assembled Genomes of Pig Fecal Samples in Nine European Countries: Insights into Antibiotic Resistance Genes and Viruses. Microorganisms.

[88] Liu Z., Klümper U., Liu Y., Yang Y., Wei Q., Lin J.-G., Gu J.-D., Li M. (2019). Metagenomic and Metatranscriptomic Analyses Reveal Activity and Hosts of Antibiotic Resistance Genes in Activated Sludge. Environ. Int..

[89] Ma W., Yin L., Hu Y., Liu X., Guo Z., Zhong B., Qiu H., Li J. (2024). Multi-Omics Analysis Reveals Interactions between Host and Microbes in Bama Miniature Pigs during Weaning. Front. Microbiol..

[90] Zheng X., Xu L., Tang Q., Shi K., Wang Z., Shi L., Ding Y., Yin Z., Zhang X. (2024). Integrated Metagenomic and Metabolomics Profiling Reveals Key Gut Microbiota and Metabolites Associated with Weaning Stress in Piglets. Genes.

[91] Onarman Umu Ö.C., Mydland L.T., Chen C., Pérez de Nanclares M., Shurson G.C., Urriola P.E., Sørum H., Øverland M. (2024). Integrated Multi-Omics Approach Reveals Novel Associations in the Rapeseed Diet-Microbiota-Host Axis in Pigs. ISME Commun..

